# Correction: Hsf1 and the molecular chaperone Hsp90 support a ‘rewiring stress response’ leading to an adaptive cell size increase in chronic stress

**DOI:** 10.7554/eLife.107055

**Published:** 2025-04-03

**Authors:** Samarpan Maiti, Kaushik Bhattacharya, Diana Wider, Dina Hany, Olesya Panasenko, Lilia Bernasconi, Nicolas Hulo, Didier Picard

**Keywords:** Human

Maiti S, Bhattacharya K, Wider D, Hany D, Panasenko O, Bernasconi L, Hulo N, Picard D. 2023. Hsf1 and the molecular chaperone Hsp90 support a ‘rewiring stress response’ leading to an adaptive cell size increase in chronic stress. *eLife* **12**:RP88658. doi: 10.7554/eLife.88658.

Published 7 December 2023

We have recently become aware of some mistakes that happened when we prepared the source files. While none of them change anything about the figures or the text nor any of the conclusions of the paper itself, we prefer to point them out and to correct them as part of an Erratum. We apologize for any inconvenience these mistakes may have caused.

The following three source data files need to be corrected:

(1) Figure 6 – source data 2: The panels for the p-S6 and S6 immunoblots were swapped for the Hsp90α KO and Hsp90β KO samples in this file. The names of the accompanying raw blots were corrected accordingly. Figure 6 itself is correct as published.

The relevant section of the corrected Figure 6 – source data 2 figure is shown here:

**Figure 1:**
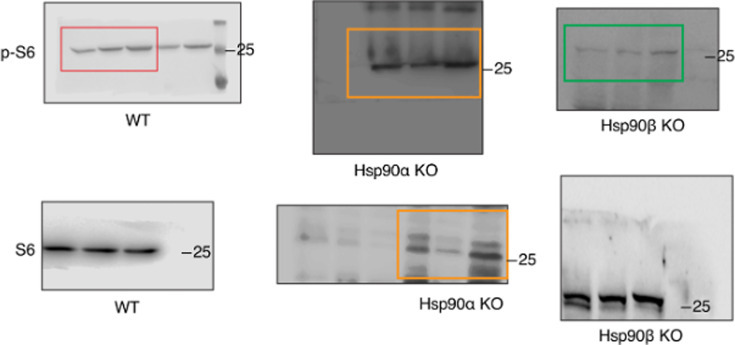


The same section of the originally published Figure 6 – source data 2 figure is shown for reference:

**Figure 2:**
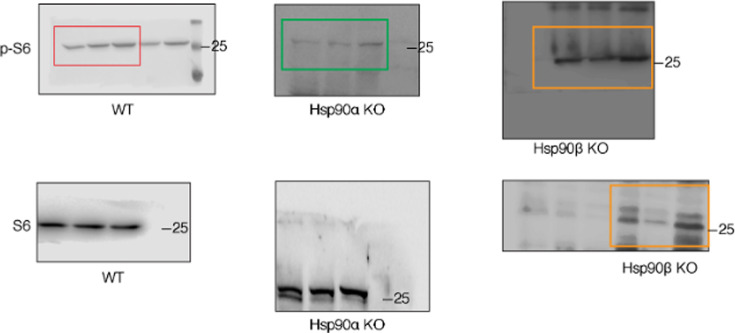


(2) Figure 6 – figure supplement 2 – source data 1: An explanation for the bottom left panel of the source data 1 file was added, and the orange box at the bottom right of the source data 1 file was corrected to encompass only 4 lanes. Figure 6 – figure supplement 2 itself is correct as published.

**Figure 3:**
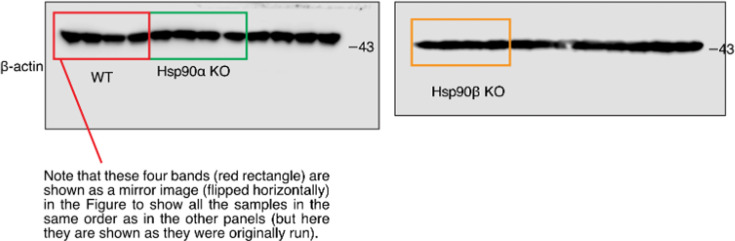


The relevant section of the corrected Figure 6 – figure supplement 2 – source data 1 figure is shown here:

**Figure 4:**
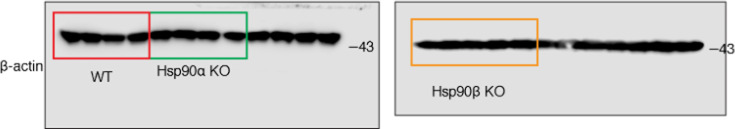


The same section of the originally published Figure 6 – figure supplement 2 – source data 1 figure is shown for reference:

Figure 9 – source data 1: the labels below the second and third immunoblots were swapped in this figure. Figure 9 itself is correct as published.

The relevant section of the corrected Figure 9 – source data 1 figure is shown here:

**Figure 5:**
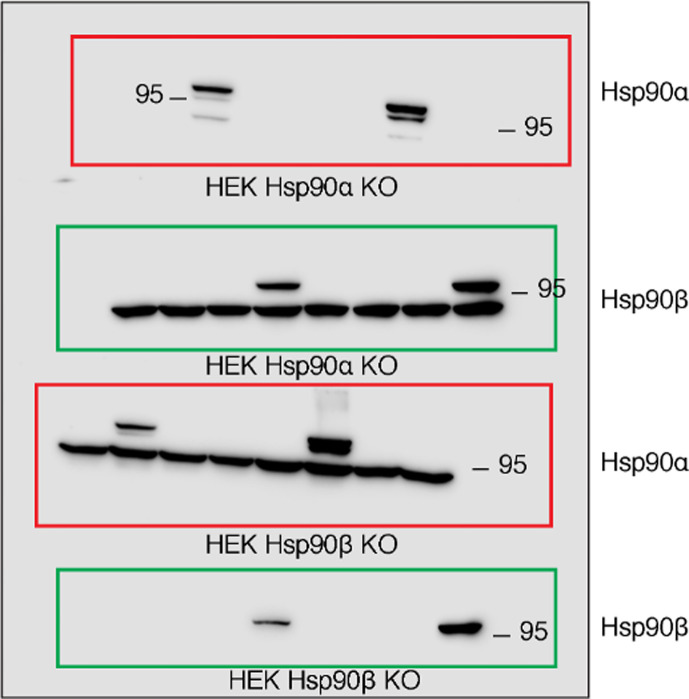


The same section of the originally published Figure 9 – source data 1 figure is shown for reference:

**Figure 6:**
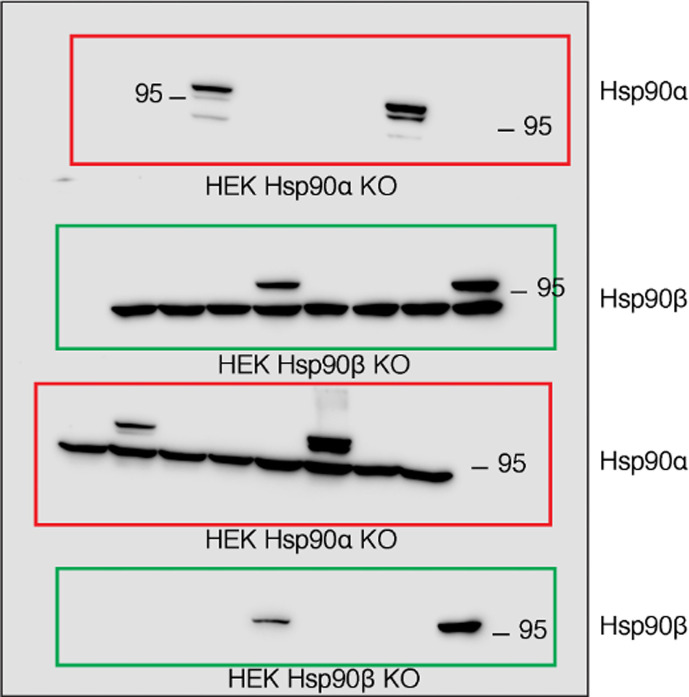


The article has been corrected accordingly.

