# Supplementary figures and images for: Hsf1 and the molecular chaperone Hsp90 support a ‘rewiring stress response’ leading to an adaptive cell size increase in chronic stress
Source: eLife. 2023 Dec 7;12:RP88658. doi: 10.7554/eLife.88658 (PMC10703448; doi:10.7554/eLife.88658)

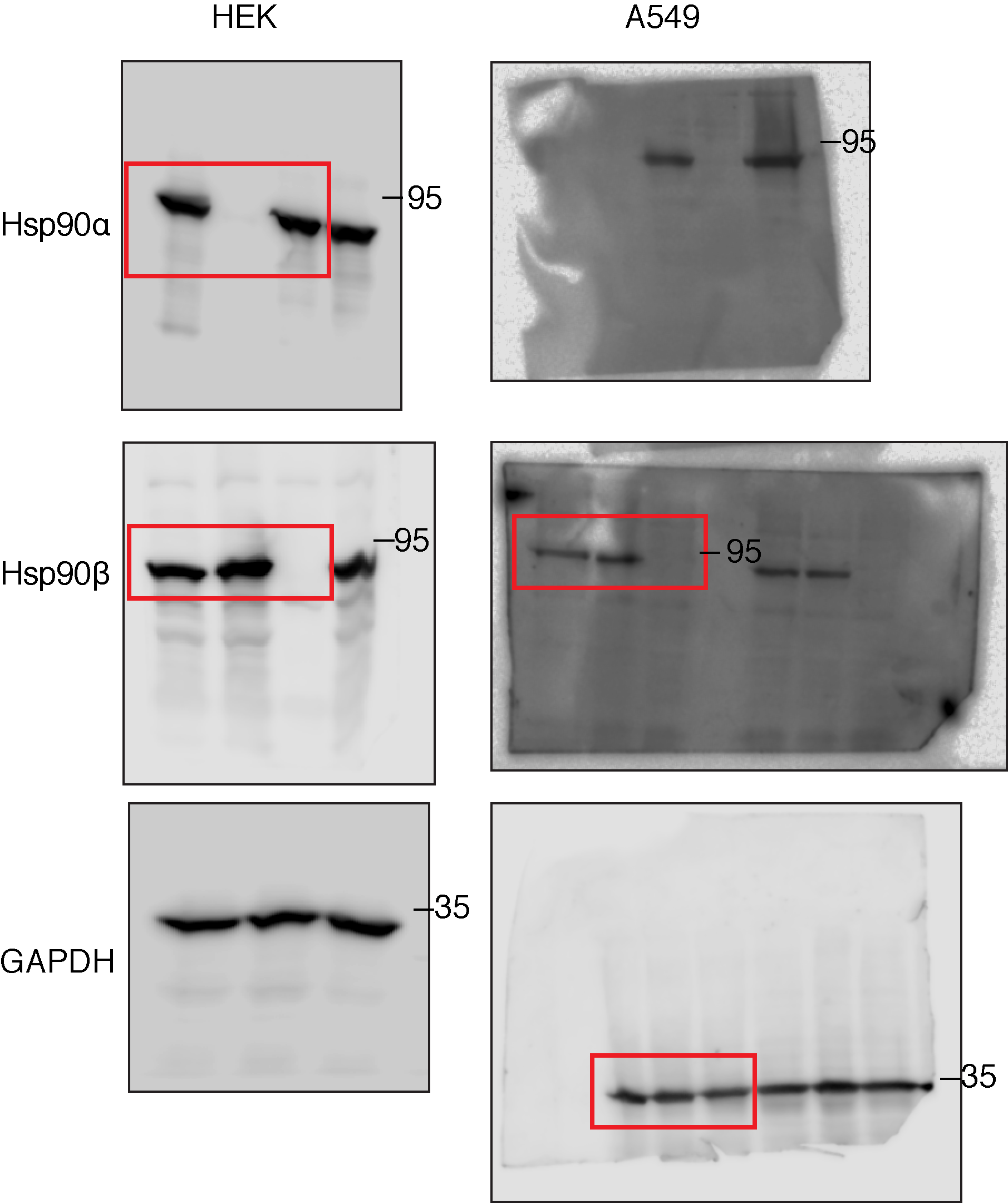

Supplement: Figure 2—source data 1. [file elife-88658-fig2-data1.zip › Fig2-source data 1/Fig2-source data 1.png]

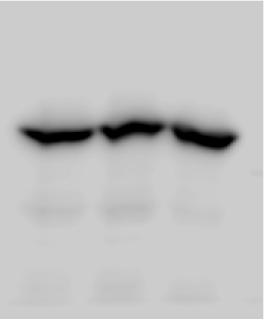

Supplement: Figure 2—source data 1. [file elife-88658-fig2-data1.zip › Fig2-source data 1/raw blots/actin 1.tif]

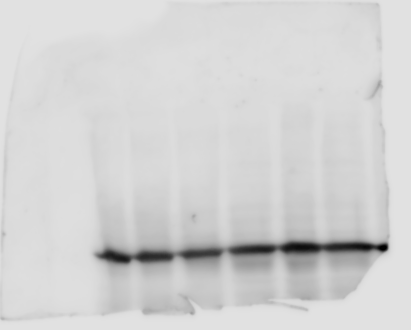

Supplement: Figure 2—source data 1. [file elife-88658-fig2-data1.zip › Fig2-source data 1/raw blots/actin-2.tif]

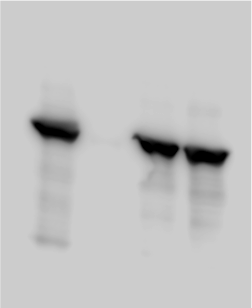

Supplement: Figure 2—source data 1. [file elife-88658-fig2-data1.zip › Fig2-source data 1/raw blots/Hsp90a 1.tif]

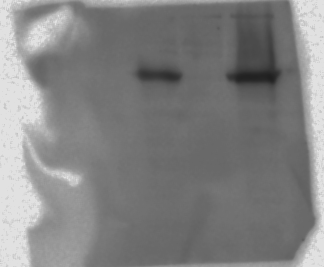

Supplement: Figure 2—source data 1. [file elife-88658-fig2-data1.zip › Fig2-source data 1/raw blots/90a-2.tif]

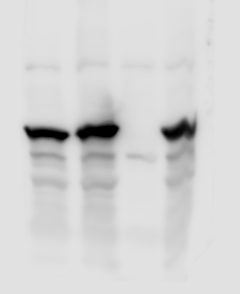

Supplement: Figure 2—source data 1. [file elife-88658-fig2-data1.zip › Fig2-source data 1/raw blots/90b 2.tif]

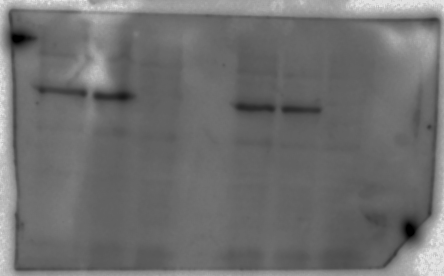

Supplement: Figure 2—source data 1. [file elife-88658-fig2-data1.zip › Fig2-source data 1/raw blots/90b-1.tif]

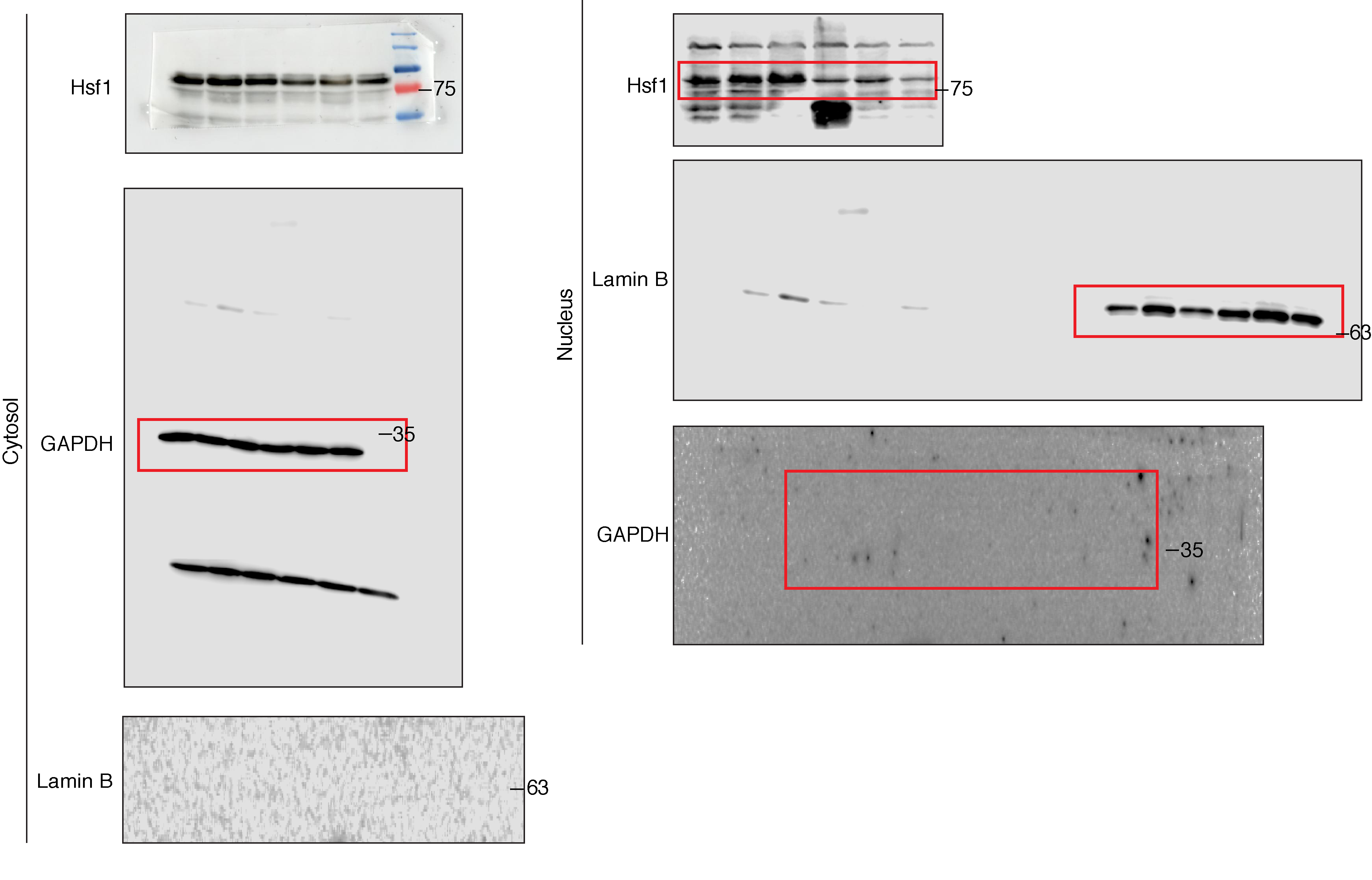

Supplement: Figure 3—source data 2. [file elife-88658-fig3-data2.zip › Fig3-source data 2/Fig3-source data 2.png]

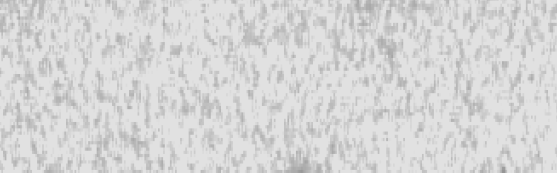

Supplement: Figure 3—source data 2. [file elife-88658-fig3-data2.zip › Fig3-source data 2/raw blots/Lamin B-1.tif]

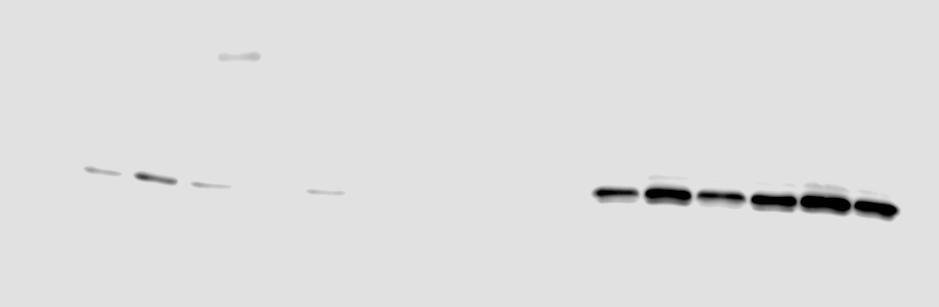

Supplement: Figure 3—source data 2. [file elife-88658-fig3-data2.zip › Fig3-source data 2/raw blots/Lamin B-2.tif]

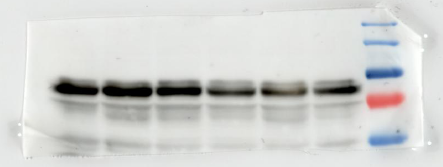

Supplement: Figure 3—source data 2. [file elife-88658-fig3-data2.zip › Fig3-source data 2/raw blots/Hsf1 -1.tif]

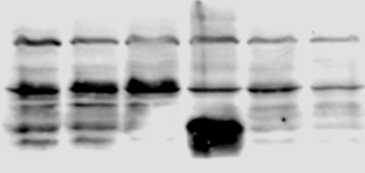

Supplement: Figure 3—source data 2. [file elife-88658-fig3-data2.zip › Fig3-source data 2/raw blots/Hsf1 -2.tif]

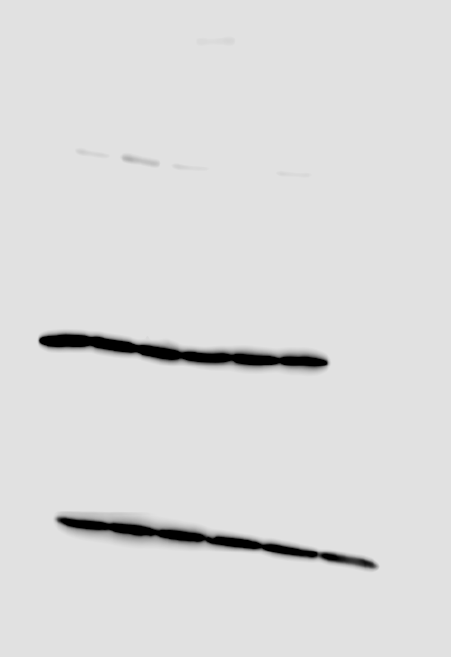

Supplement: Figure 3—source data 2. [file elife-88658-fig3-data2.zip › Fig3-source data 2/raw blots/GAPDH-1.tif]

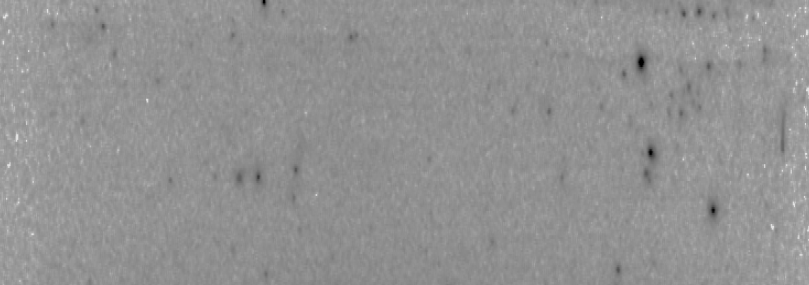

Supplement: Figure 3—source data 2. [file elife-88658-fig3-data2.zip › Fig3-source data 2/raw blots/GAPDH-2.tif]

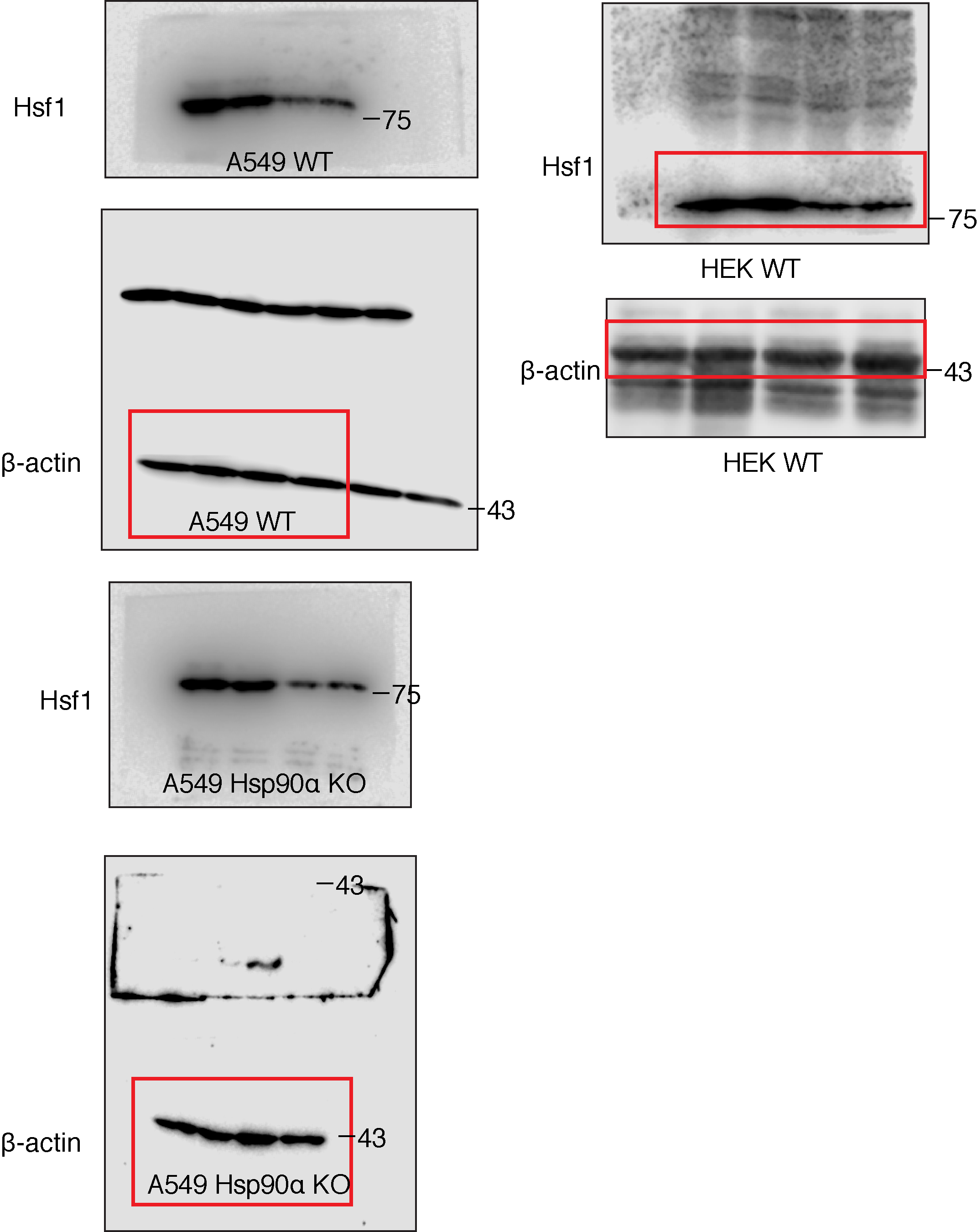

Supplement: Figure 3—figure supplement 1—source data 2. [file elife-88658-fig3-figsupp1-data2.zip › Fig3-FigSuppl1-source data 2/Fig3-FigSuppl1-source data 2.png]

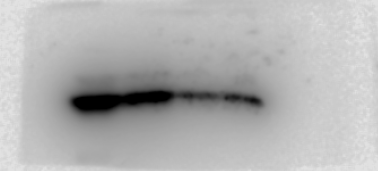

Supplement: Figure 3—figure supplement 1—source data 2. [file elife-88658-fig3-figsupp1-data2.zip › Fig3-FigSuppl1-source data 2/raw blots/Hsf1 -1.tif]

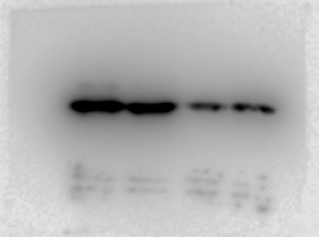

Supplement: Figure 3—figure supplement 1—source data 2. [file elife-88658-fig3-figsupp1-data2.zip › Fig3-FigSuppl1-source data 2/raw blots/Hsf1 -2.tif]

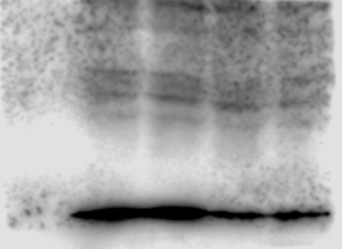

Supplement: Figure 3—figure supplement 1—source data 2. [file elife-88658-fig3-figsupp1-data2.zip › Fig3-FigSuppl1-source data 2/raw blots/Hsf1 -3.tif]

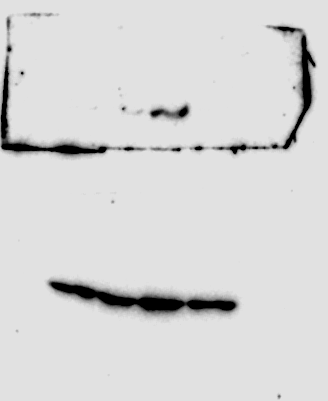

Supplement: Figure 3—figure supplement 1—source data 2. [file elife-88658-fig3-figsupp1-data2.zip › Fig3-FigSuppl1-source data 2/raw blots/╬▓-actin 3.tif]

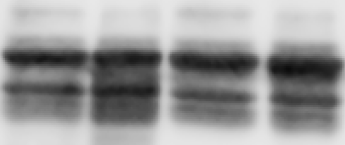

Supplement: Figure 3—figure supplement 1—source data 2. [file elife-88658-fig3-figsupp1-data2.zip › Fig3-FigSuppl1-source data 2/raw blots/╬▓-actin 2.tif]

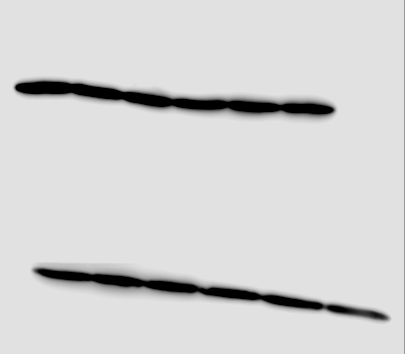

Supplement: Figure 3—figure supplement 1—source data 2. [file elife-88658-fig3-figsupp1-data2.zip › Fig3-FigSuppl1-source data 2/raw blots/╬▓-actin 1.tif]

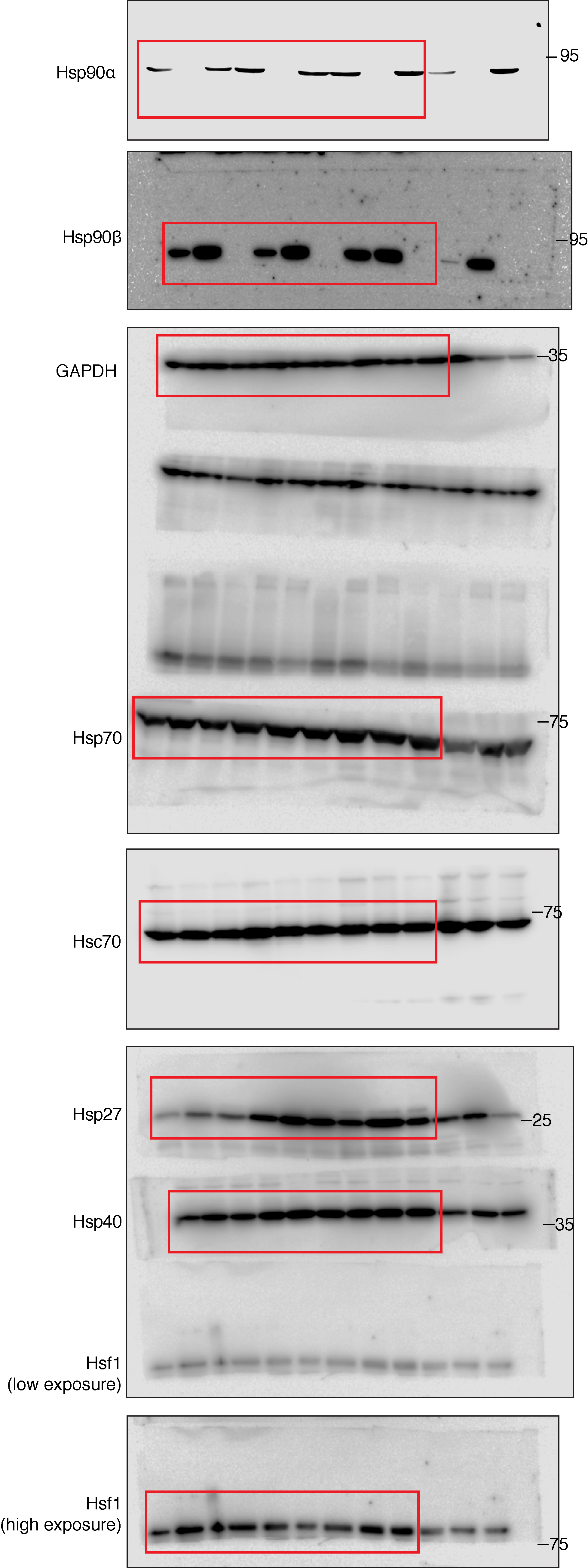

Supplement: Figure 4—source data 1. [file elife-88658-fig4-data1.zip › Fig4-source data 1/Fig4-source data 1.png]

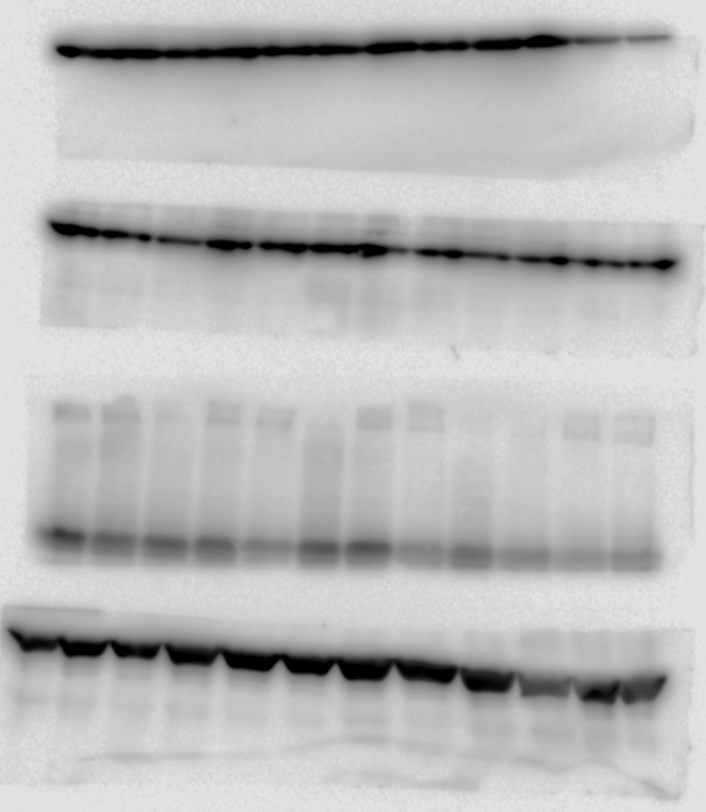

Supplement: Figure 4—source data 1. [file elife-88658-fig4-data1.zip › Fig4-source data 1/raw blots/GAPDH- Hsp70.tif]

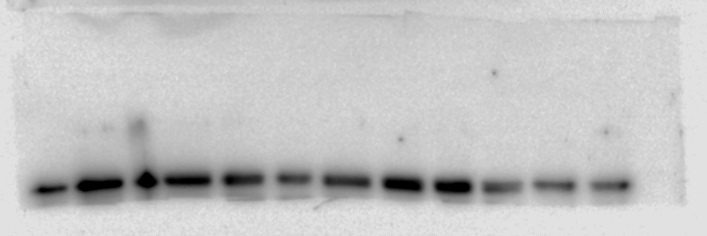

Supplement: Figure 4—source data 1. [file elife-88658-fig4-data1.zip › Fig4-source data 1/raw blots/Hsf1.tif]

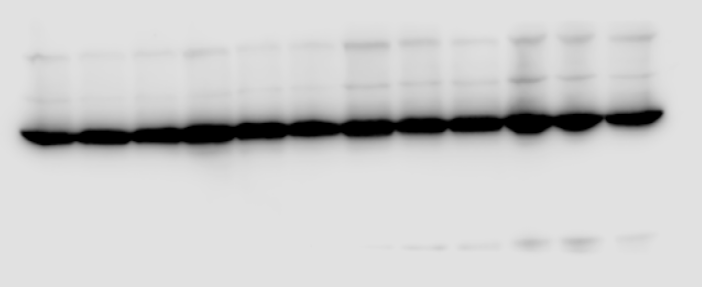

Supplement: Figure 4—source data 1. [file elife-88658-fig4-data1.zip › Fig4-source data 1/raw blots/Hsc70.tif]

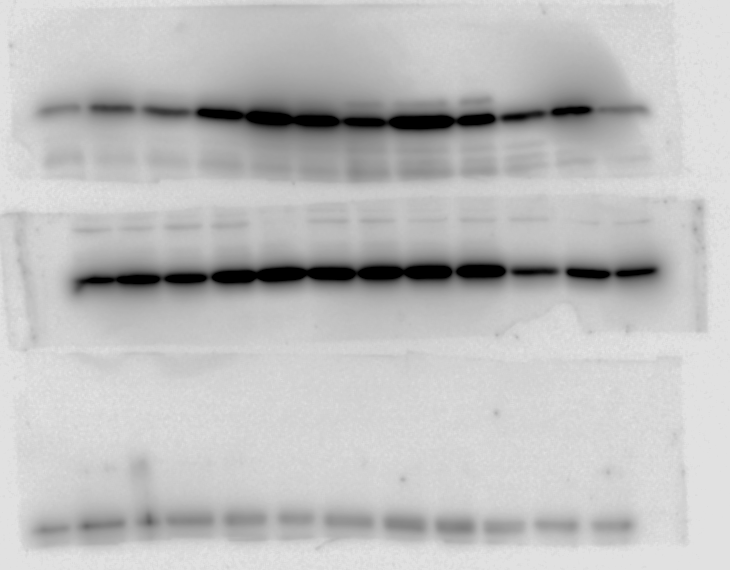

Supplement: Figure 4—source data 1. [file elife-88658-fig4-data1.zip › Fig4-source data 1/raw blots/Hsp27- Hsp40.tif]

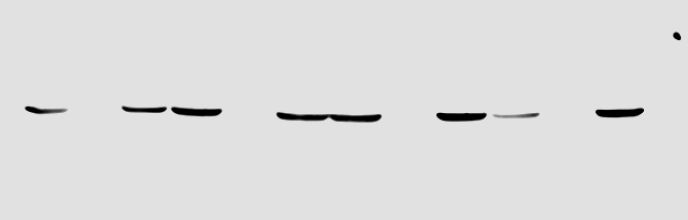

Supplement: Figure 4—source data 1. [file elife-88658-fig4-data1.zip › Fig4-source data 1/raw blots/Hsp90╬▒.tif]

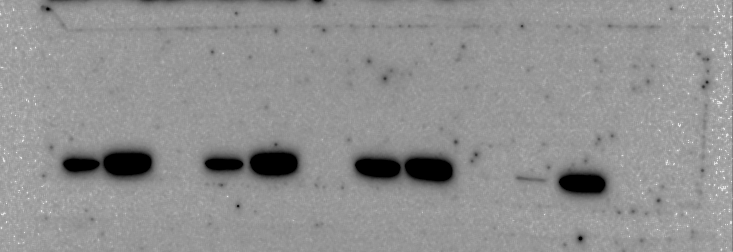

Supplement: Figure 4—source data 1. [file elife-88658-fig4-data1.zip › Fig4-source data 1/raw blots/Hsp90╬▓.tif]

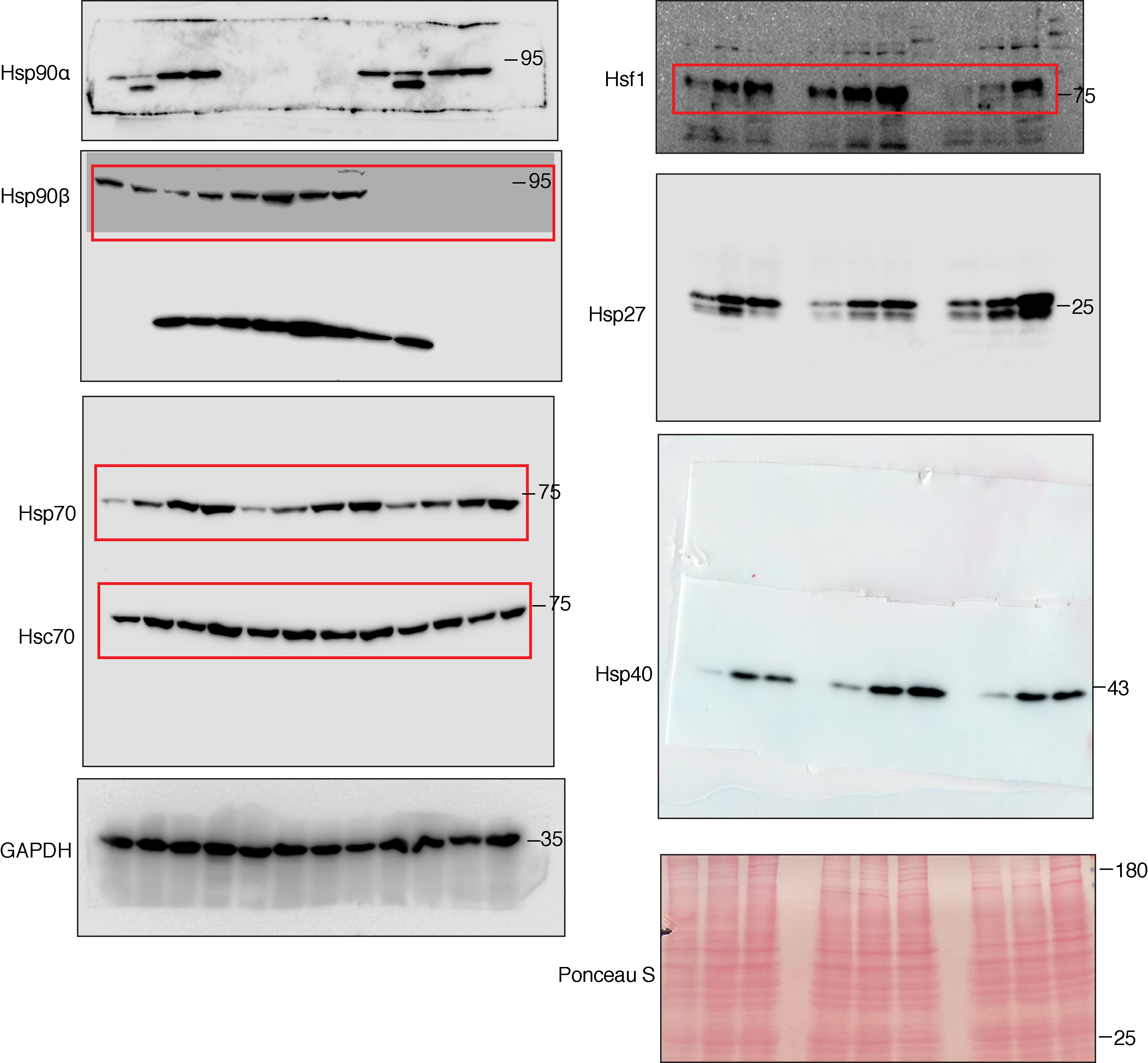

Supplement: Figure 4—figure supplement 1—source data 1. [file elife-88658-fig4-figsupp1-data1.zip › Fig4-FigSuppl1-source data 1/Fig4-FigSuppl1-source data 1.png]

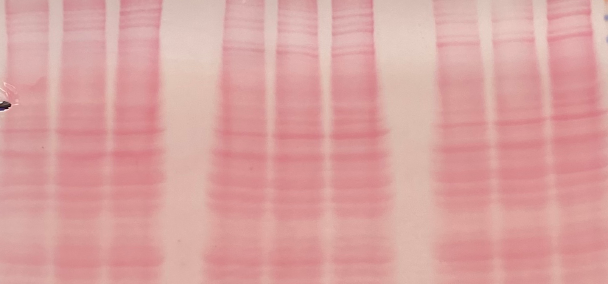

Supplement: Figure 4—figure supplement 1—source data 1. [file elife-88658-fig4-figsupp1-data1.zip › Fig4-FigSuppl1-source data 1/raw blots/Ponceau S.tif]

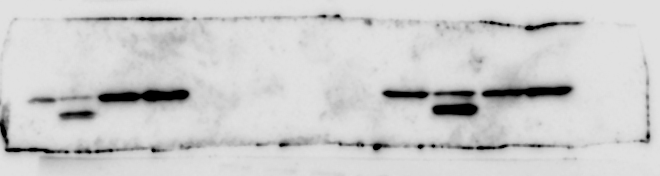

Supplement: Figure 4—figure supplement 1—source data 1. [file elife-88658-fig4-figsupp1-data1.zip › Fig4-FigSuppl1-source data 1/raw blots/Hsp90╬▒ .tif]

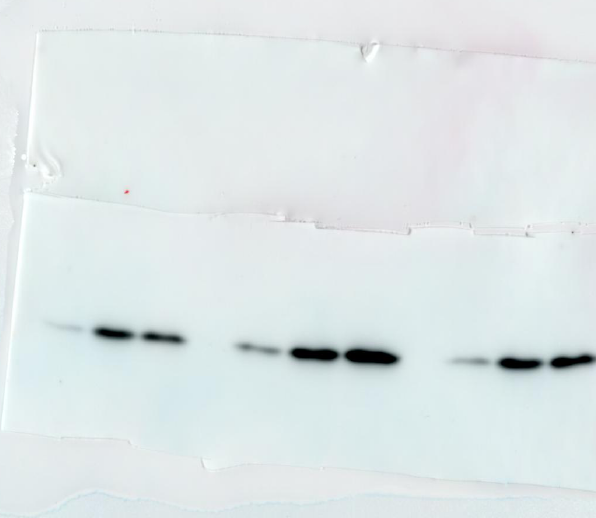

Supplement: Figure 4—figure supplement 1—source data 1. [file elife-88658-fig4-figsupp1-data1.zip › Fig4-FigSuppl1-source data 1/raw blots/Hsp40 .tif]

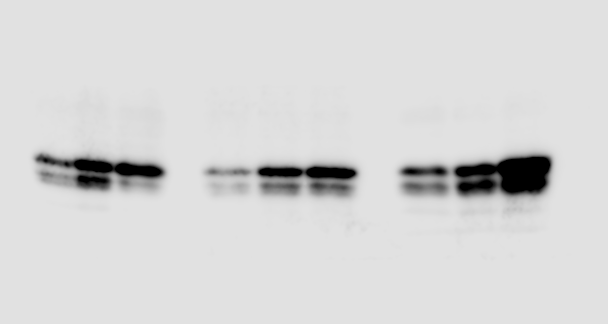

Supplement: Figure 4—figure supplement 1—source data 1. [file elife-88658-fig4-figsupp1-data1.zip › Fig4-FigSuppl1-source data 1/raw blots/Hsp27 .tif]

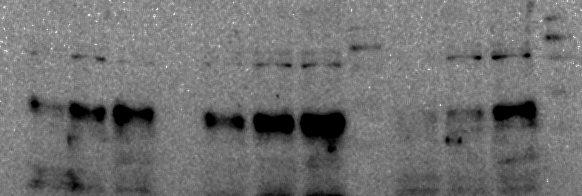

Supplement: Figure 4—figure supplement 1—source data 1. [file elife-88658-fig4-figsupp1-data1.zip › Fig4-FigSuppl1-source data 1/raw blots/Hsf1 .tif]

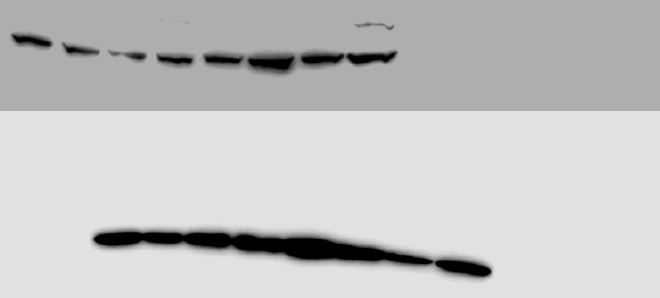

Supplement: Figure 4—figure supplement 1—source data 1. [file elife-88658-fig4-figsupp1-data1.zip › Fig4-FigSuppl1-source data 1/raw blots/Hsp90╬▓.tif]

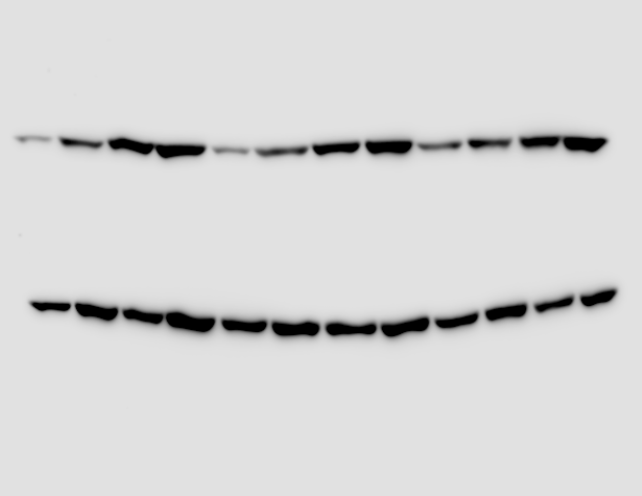

Supplement: Figure 4—figure supplement 1—source data 1. [file elife-88658-fig4-figsupp1-data1.zip › Fig4-FigSuppl1-source data 1/raw blots/Hsp70 - hsc70.tif]

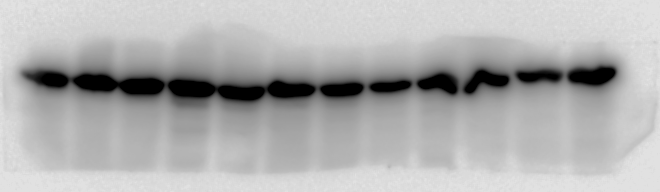

Supplement: Figure 4—figure supplement 1—source data 1. [file elife-88658-fig4-figsupp1-data1.zip › Fig4-FigSuppl1-source data 1/raw blots/GAPDH .tif]

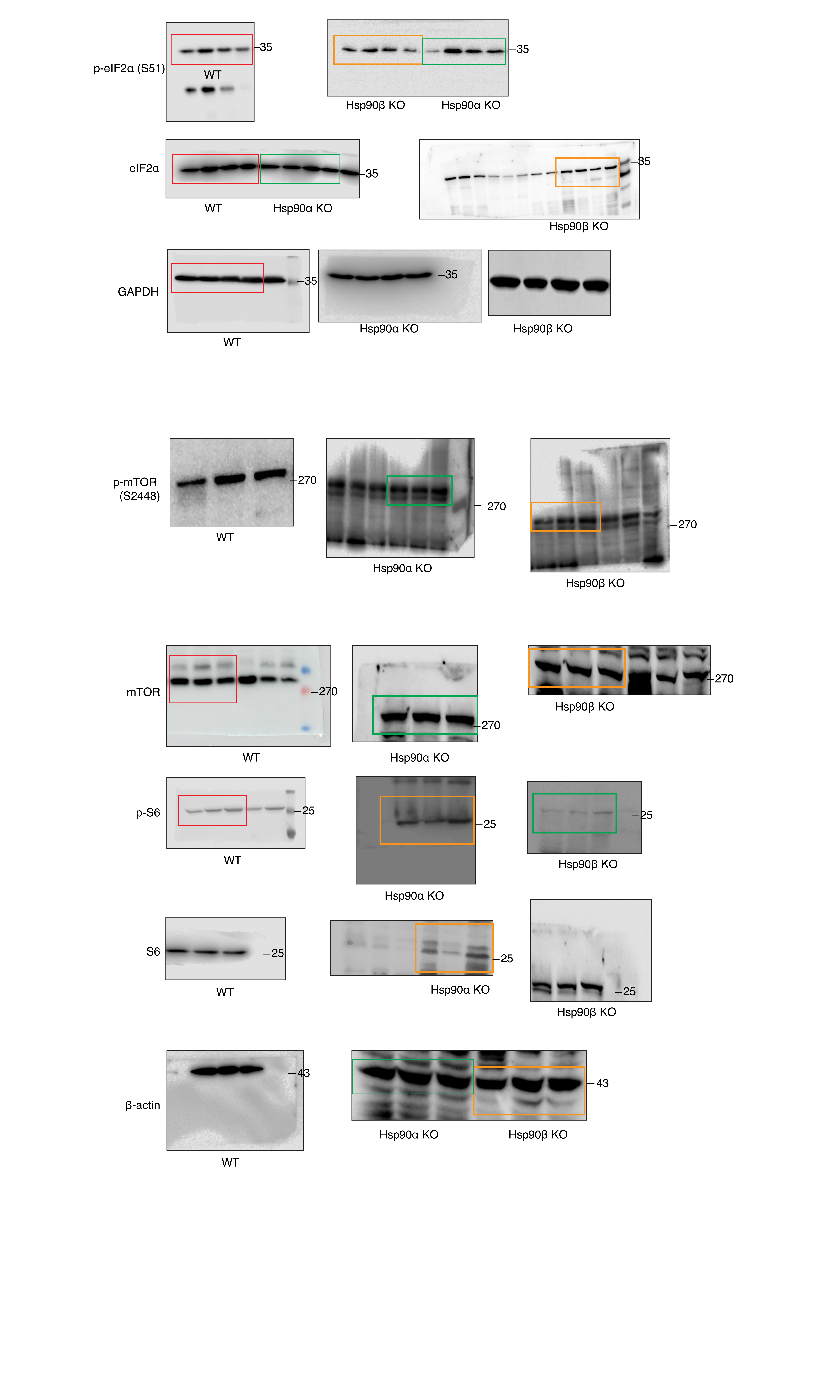

Supplement: Figure 6—source data 2. [file elife-88658-fig6-data2.zip › Fig6-source data 2.png]

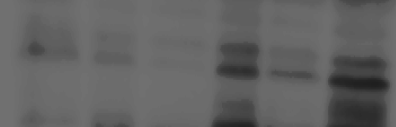

Supplement: Figure 6—source data 2. [file elife-88658-fig6-data2.zip › raw blots/S6-2.tif]

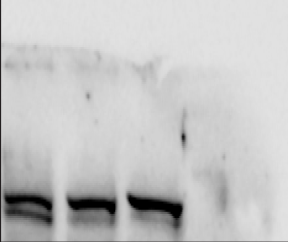

Supplement: Figure 6—source data 2. [file elife-88658-fig6-data2.zip › raw blots/S6-3.tif]

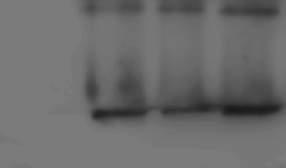

Supplement: Figure 6—source data 2. [file elife-88658-fig6-data2.zip › raw blots/p-S6 -2.tif]

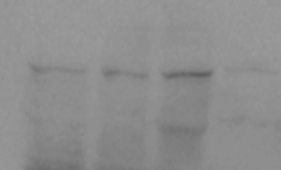

Supplement: Figure 6—source data 2. [file elife-88658-fig6-data2.zip › raw blots/p-S6 -3.tif]

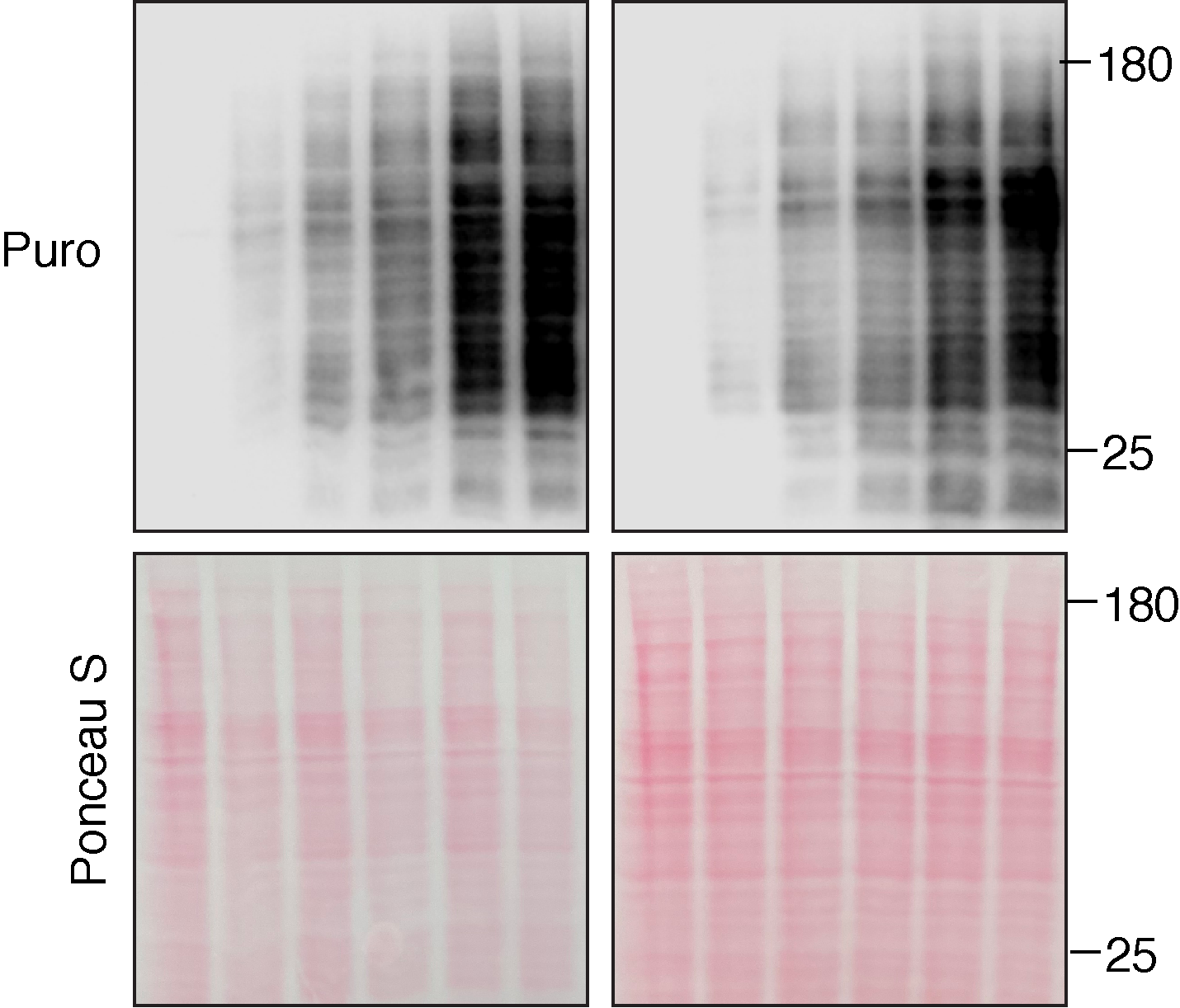

Supplement: Figure 6—figure supplement 1—source data 2. [file elife-88658-fig6-figsupp1-data2.zip › Fig6-FigSuppl1-source data 2/Fig6-FigSuppl1-source data 2.png]

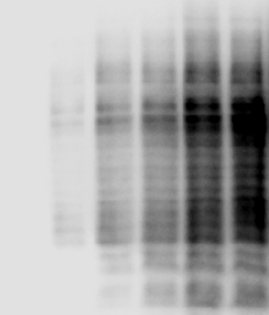

Supplement: Figure 6—figure supplement 1—source data 2. [file elife-88658-fig6-figsupp1-data2.zip › Fig6-FigSuppl1-source data 2/raw blots/Puro-2.tif]

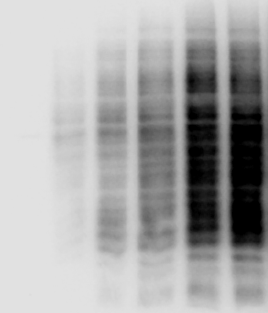

Supplement: Figure 6—figure supplement 1—source data 2. [file elife-88658-fig6-figsupp1-data2.zip › Fig6-FigSuppl1-source data 2/raw blots/Puro-1.tif]

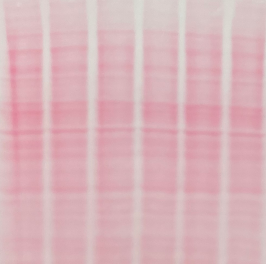

Supplement: Figure 6—figure supplement 1—source data 2. [file elife-88658-fig6-figsupp1-data2.zip › Fig6-FigSuppl1-source data 2/raw blots/Ponceau S-2.tif]

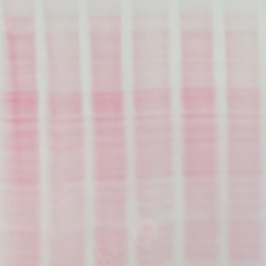

Supplement: Figure 6—figure supplement 1—source data 2. [file elife-88658-fig6-figsupp1-data2.zip › Fig6-FigSuppl1-source data 2/raw blots/Ponceau S-1.tif]

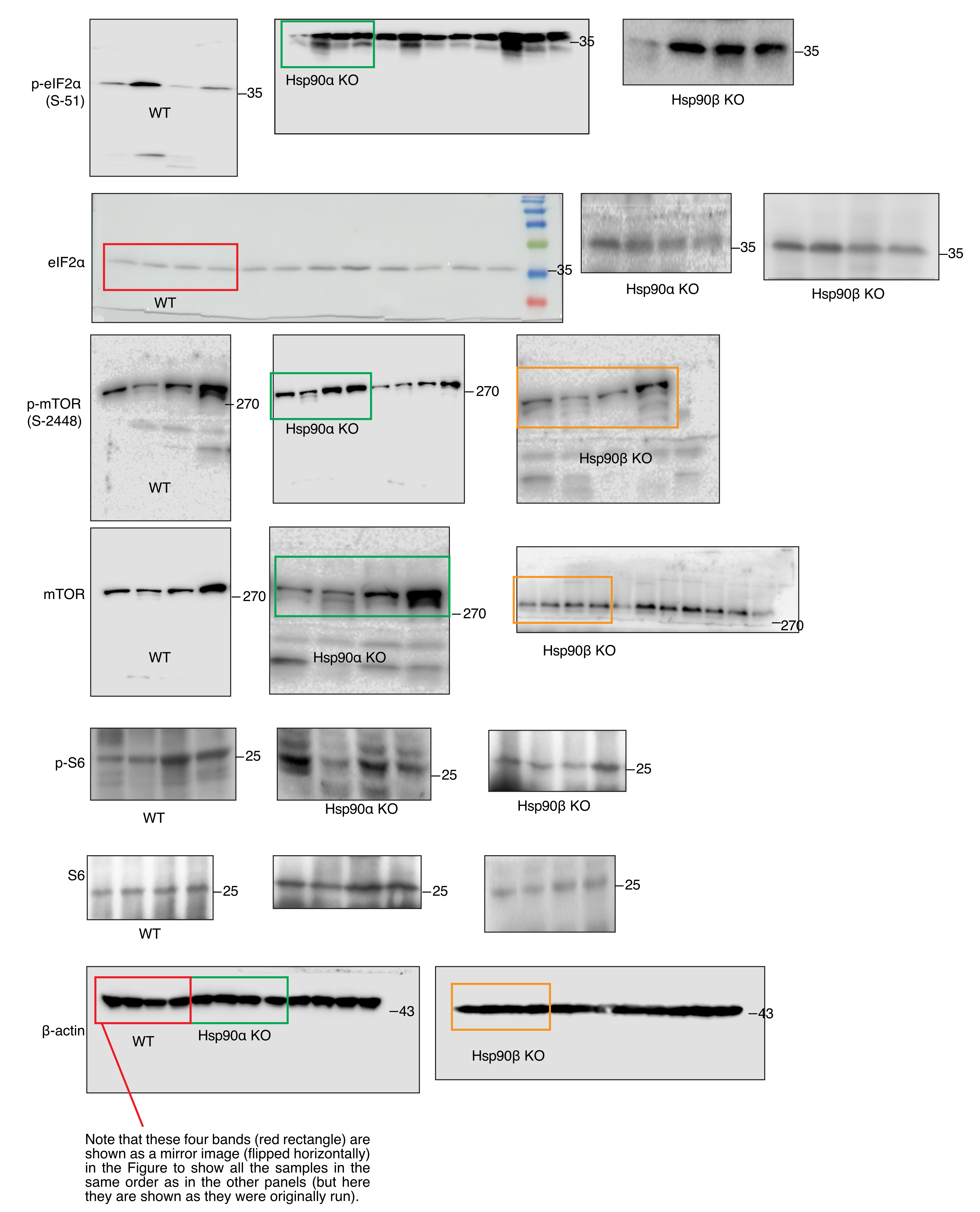

Supplement: Figure 6—figure supplement 2—source data 1. [file elife-88658-fig6-figsupp2-data1.zip › Fig6-FigSuppl2-source data 1.png]

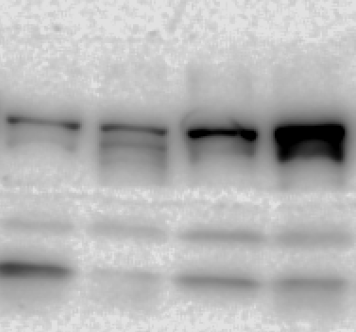

Supplement: Figure 6—figure supplement 2—source data 1. [file elife-88658-fig6-figsupp2-data1.zip › raw blots/mTOR 2.tif]

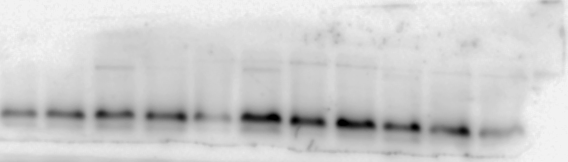

Supplement: Figure 6—figure supplement 2—source data 1. [file elife-88658-fig6-figsupp2-data1.zip › raw blots/mTOR 3.tif]

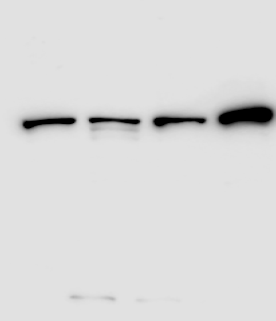

Supplement: Figure 6—figure supplement 2—source data 1. [file elife-88658-fig6-figsupp2-data1.zip › raw blots/mTOR 1.tif]

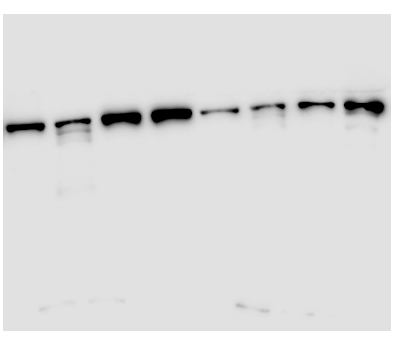

Supplement: Figure 6—figure supplement 2—source data 1. [file elife-88658-fig6-figsupp2-data1.zip › raw blots/p-mTOR 2.tif]

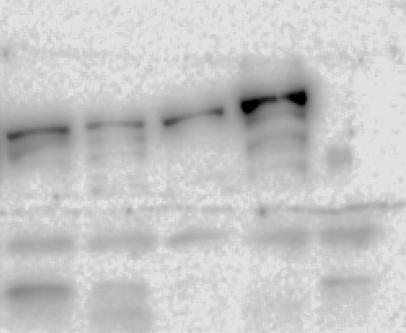

Supplement: Figure 6—figure supplement 2—source data 1. [file elife-88658-fig6-figsupp2-data1.zip › raw blots/p-mTOR 3.tif]

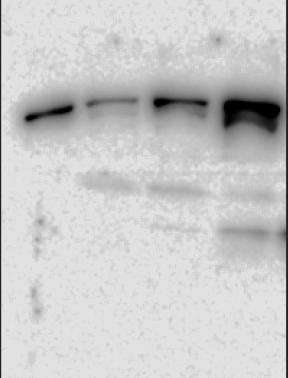

Supplement: Figure 6—figure supplement 2—source data 1. [file elife-88658-fig6-figsupp2-data1.zip › raw blots/p-mTOR 1.tif]

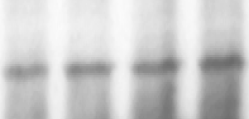

Supplement: Figure 6—figure supplement 2—source data 1. [file elife-88658-fig6-figsupp2-data1.zip › raw blots/S6 1.tif]

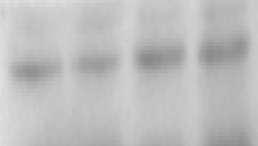

Supplement: Figure 6—figure supplement 2—source data 1. [file elife-88658-fig6-figsupp2-data1.zip › raw blots/S6 3.tif]

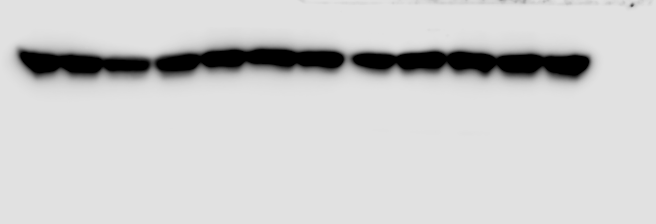

Supplement: Figure 6—figure supplement 2—source data 1. [file elife-88658-fig6-figsupp2-data1.zip › raw blots/╬▓-actin 1 and 2.tif]

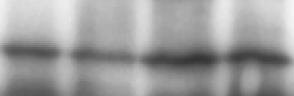

Supplement: Figure 6—figure supplement 2—source data 1. [file elife-88658-fig6-figsupp2-data1.zip › raw blots/S6 2.tif]

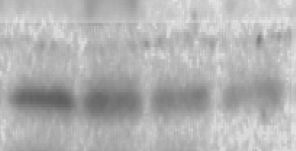

Supplement: Figure 6—figure supplement 2—source data 1. [file elife-88658-fig6-figsupp2-data1.zip › raw blots/eIF2╬▒ 2.tif]

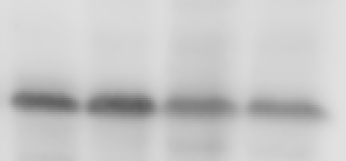

Supplement: Figure 6—figure supplement 2—source data 1. [file elife-88658-fig6-figsupp2-data1.zip › raw blots/eIF2╬▒ 3.tif]

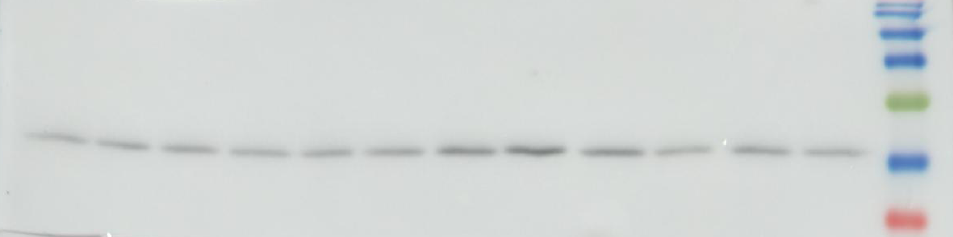

Supplement: Figure 6—figure supplement 2—source data 1. [file elife-88658-fig6-figsupp2-data1.zip › raw blots/eIF2╬▒ 1.tif]

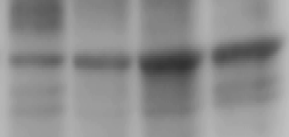

Supplement: Figure 6—figure supplement 2—source data 1. [file elife-88658-fig6-figsupp2-data1.zip › raw blots/p-S6 1.tif]

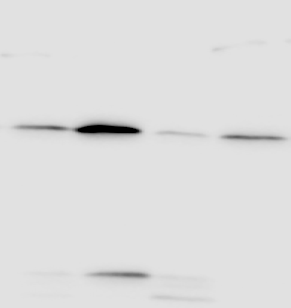

Supplement: Figure 6—figure supplement 2—source data 1. [file elife-88658-fig6-figsupp2-data1.zip › raw blots/p-eIF2╬▒ 1.tif]

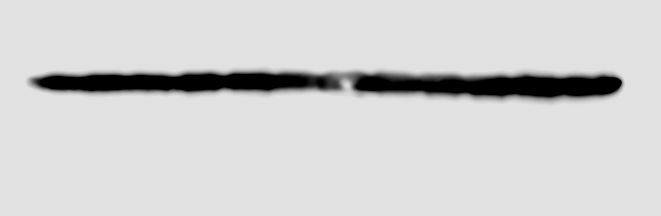

Supplement: Figure 6—figure supplement 2—source data 1. [file elife-88658-fig6-figsupp2-data1.zip › raw blots/╬▓-actin 3.tif]

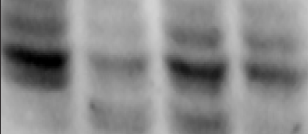

Supplement: Figure 6—figure supplement 2—source data 1. [file elife-88658-fig6-figsupp2-data1.zip › raw blots/p-S6 2.tif]

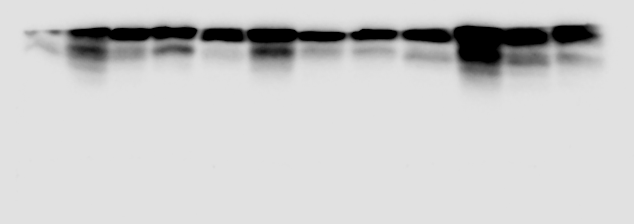

Supplement: Figure 6—figure supplement 2—source data 1. [file elife-88658-fig6-figsupp2-data1.zip › raw blots/p-eIF2╬▒ 2.tif]

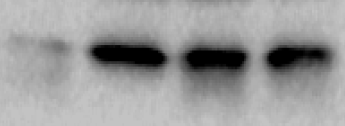

Supplement: Figure 6—figure supplement 2—source data 1. [file elife-88658-fig6-figsupp2-data1.zip › raw blots/p-eIF2╬▒ 3.tif]

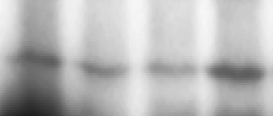

Supplement: Figure 6—figure supplement 2—source data 1. [file elife-88658-fig6-figsupp2-data1.zip › raw blots/p-S6 3.tif]

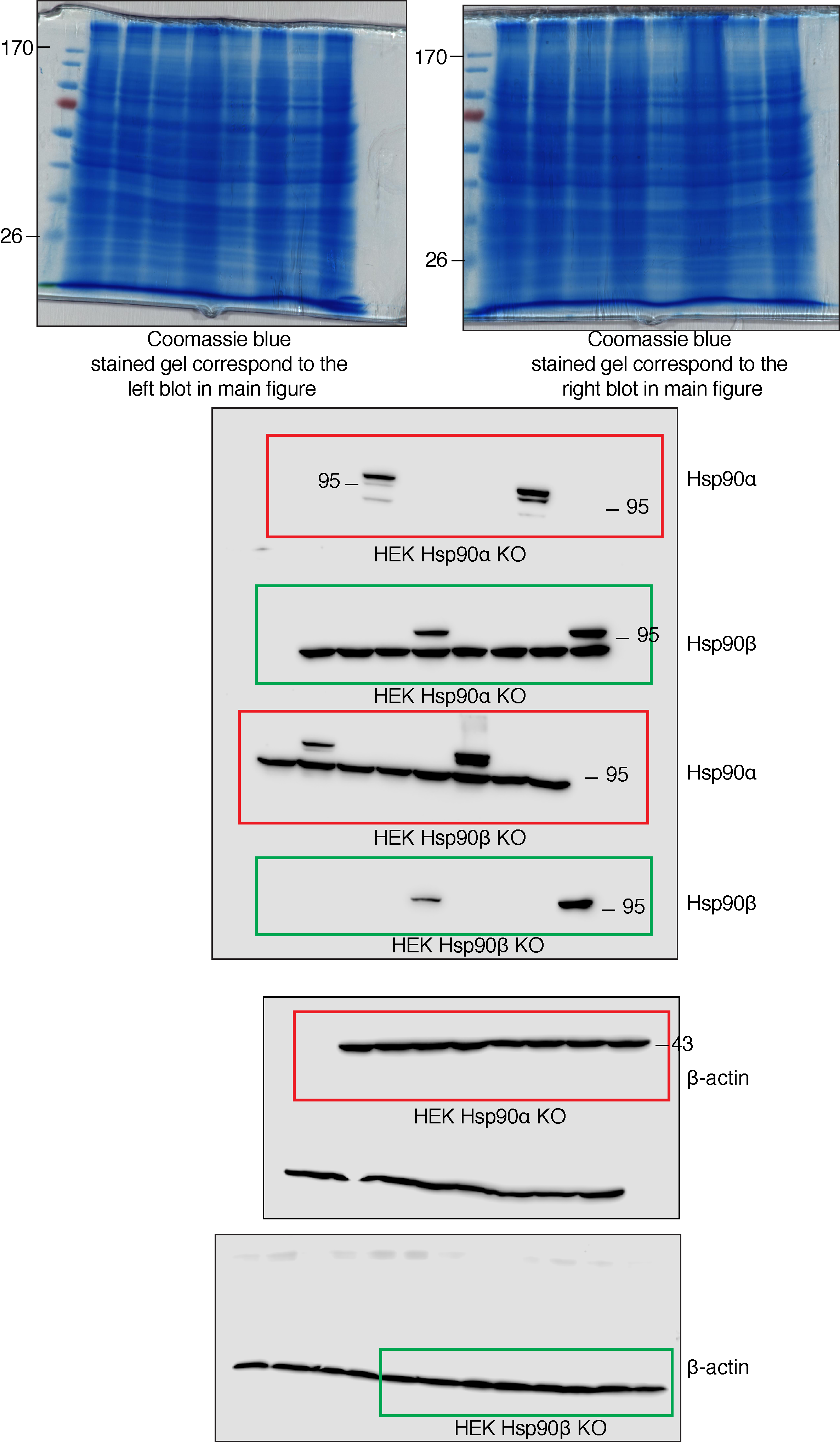

Supplement: Figure 9—source data 1. [file elife-88658-fig9-data1.zip › Fig9-source data 1.png]

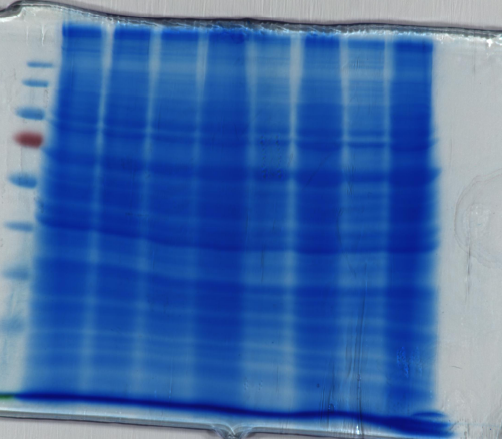

Supplement: Figure 9—source data 1. [file elife-88658-fig9-data1.zip › raw blots/Coomassie blue gel 1.tif]

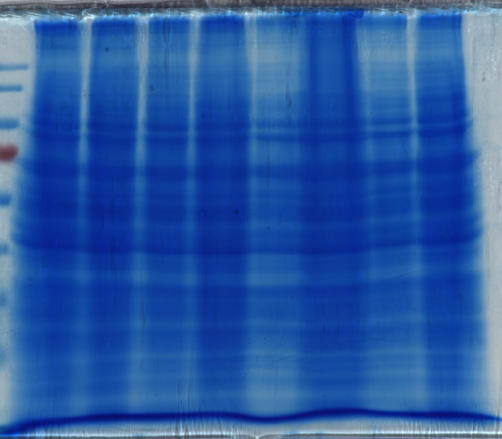

Supplement: Figure 9—source data 1. [file elife-88658-fig9-data1.zip › raw blots/Coomassie blue gel 2.tif]

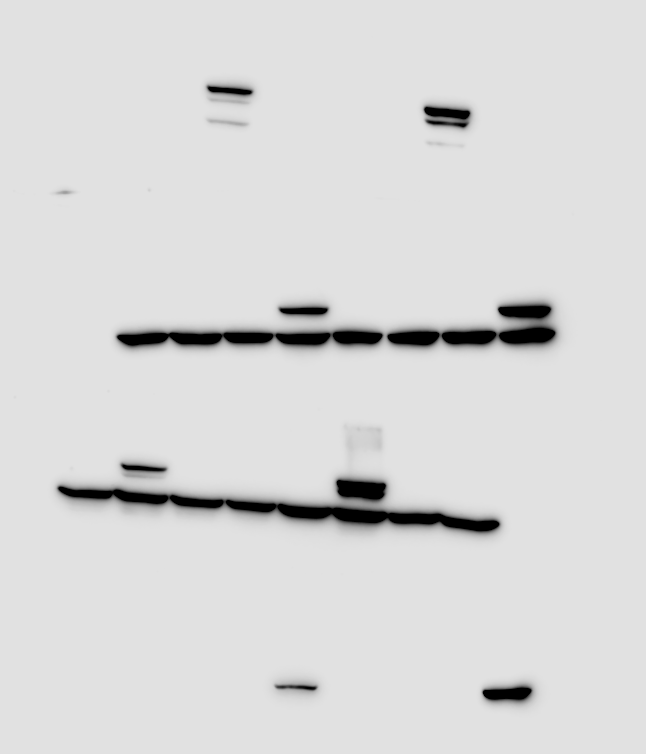

Supplement: Figure 9—source data 1. [file elife-88658-fig9-data1.zip › raw blots/Hsp90╬▒ : Hsp90╬▓ .tif]

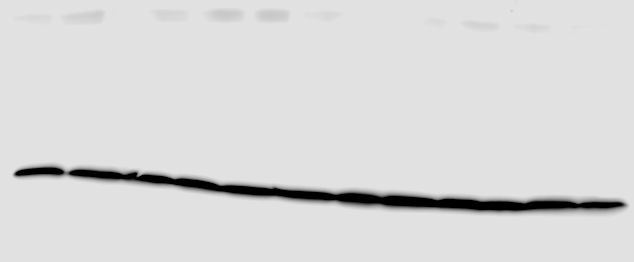

Supplement: Figure 9—source data 1. [file elife-88658-fig9-data1.zip › raw blots/╬▓-actin 2.tif]

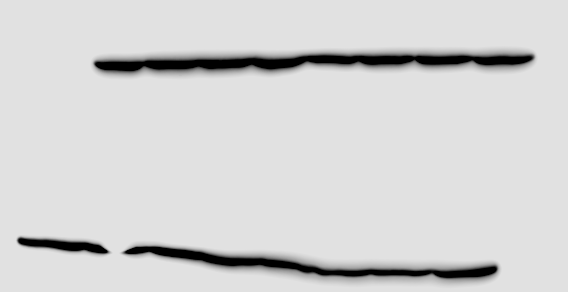

Supplement: Figure 9—source data 1. [file elife-88658-fig9-data1.zip › raw blots/╬▓-actin 1.tif]
